# Maternal and infant outcomes in sarcoidosis pregnancy: a Swedish population-based cohort study of first births

**DOI:** 10.1186/s12931-020-01493-y

**Published:** 2020-08-27

**Authors:** Laura Köcher, Marios Rossides, Katarina Remaeus, Johan Grunewald, Anders Eklund, Susanna Kullberg, Elizabeth V. Arkema

**Affiliations:** 1 Clinical Epidemiology Division, T2, Department of Medicine Solna, Karolinska Institutet, 171 76 Stockholm, Sweden; 2 Respiratory Medicine Division, Department of Medicine Solna, Karolinska Institutet, Stockholm, Sweden; 3 Center for Molecular Medicine, Karolinska Institutet, Stockholm, Sweden; 4 Department of Respiratory Medicine, Karolinska University Hospital, Stockholm, Sweden

**Keywords:** Sarcoidosis, Pregnancy, Infant, Preeclampsia, Cesarean delivery, Preterm birth, Birth defect

## Abstract

**Background:**

It is unclear whether sarcoidosis, a multisystem inflammatory disease, is associated with adverse pregnancy outcomes. We aimed to assess the risk of adverse maternal and infant outcomes in sarcoidosis pregnancies, focused on first births.

**Methods:**

Using a population-based cohort study design and Swedish national registers (2002–2013), we identified 182 singleton first pregnancies in the Medical Birth Register with at least two maternal ICD-coded sarcoidosis visits prior to pregnancy in the National Patient Register. Modified Poisson regression models estimated relative risks (RR) of adverse outcomes in sarcoidosis pregnancies compared to the general population adjusted for maternal age at delivery, calendar year and educational level. Some models were additionally adjusted for maternal body mass index and smoking status.

**Results:**

The prevalence of pre-existing diabetes and hypertension was higher in mothers with sarcoidosis than those without sarcoidosis. Mothers with sarcoidosis had an increased risk of preeclampsia/eclampsia (RR 1.6; 95%CI 1.0, 2.6) and cesarean delivery (RR 1.3; 95%CI 1.0, 1.6). There were < 5 stillbirths and cases of infection and no cases of placental abruption, venous thromboembolism, cardiac arrest or maternal death. Newborns of first-time mothers with sarcoidosis had a 70% increased risk of preterm birth (RR 1.7; 95%CI 1.1, 2.5). There was an increased risk of birth defects (RR 1.6; 95%CI 0.9, 2.8) the majority of which were non-cardiac.

**Conclusions:**

Sarcoidosis is associated with increased risks for preeclampsia/eclampsia, cesarean delivery, preterm birth and some birth defects. Awareness of these conditions may prevent possible pregnancy complications in mothers with sarcoidosis and their newborns.

## Introduction

Sarcoidosis is a multisystem inflammatory disease characterized by the formation of non-caseating granulomas of unknown etiology, primarily affecting the pulmonary and lymphatic system. About half of the sarcoidosis patient population is female, many of whom are diagnosed at a reproductive age [1]. Several systemic inflammatory disorders are associated with an increased risk of complications during pregnancy, labor and delivery compared to the general population [2–5], however few studies have examined pregnancy in sarcoidosis.

In some case series, sarcoidosis symptoms remained unchanged or improved during pregnancy, and few adverse events were observed [6, 7]. However, other case reports have described severe adverse outcomes, including maternal mortality and cardiac arrest in patients with cardiac and neurosarcoidosis [8–11]. Only one cohort study has been conducted which included 678 deliveries from the Nationwide Inpatient Sample in the U.S. [12]. Sarcoidosis was associated with an increased risk of preeclampsia, eclampsia, cesarean delivery, postpartum hemorrhage, and venous thromboembolism (VTE) and infants born to women with sarcoidosis were more likely to be premature and have intrauterine growth restriction [12]. Women with sarcoidosis were included in the study if they received a delivery-related discharge diagnosis of sarcoidosis, which may not capture all women with a history of sarcoidosis. Furthermore, parity was not taken into account, which is an important factor since several complications are more frequent in first pregnancy [13, 14].

Prospective investigations in a large, representative and contemporary sample of women with sarcoidosis are needed to develop and update recommendations for sarcoidosis pregnancy. Our aim was to estimate the risks of adverse maternal and infant outcomes in first-time pregnancies with and without sarcoidosis in a population-based cohort study in Sweden.

## Methods

### Setting and data sources

In Sweden, healthcare data is collected in national registers and a unique personal identification number can be used to link individuals’ information across registers. Prenatal and antenatal care is publicly funded, and information on prenatal, delivery and postnatal visits is captured by the Medical Birth Register (MBR), encompassing 99% of all deliveries in Sweden since 1973 [15]. Before 2008, stillbirths occurring 28 gestational weeks or later were recorded; since January 2008, stillbirths at 22 weeks or later were included. The National Patient Register (NPR) has nationwide coverage of hospitalizations since 1987 and outpatient specialist visits since 2001. The Prescribed Drug Register was introduced in July 2005 and includes information on dispensations of prescription medications. Data were available from these registers for this study through the end of 2013.

### Study design and population

In this population-based cohort study, we identified women who had a birth registered in the MBR 2002–2013 and who had at least two inpatient or outpatient visits in the NPR listing an International Classification of Disease (ICD) code for sarcoidosis (ICD-8/9135, ICD-10 D86) prior to the estimated date of last menstrual period (calculated by subtracting estimated gestational age from the delivery date). Women from the general population without sarcoidosis (Total Population Register) who were originally matched 10:1 to sarcoidosis cases on year of birth, sex and residential location [16] were included as comparators irrespective of whether their matched case was present in our analytical sample. Multifetal pregnancies were excluded because of their increased risk of obstetric complications and adverse neonatal outcomes [17]. We restricted to first pregnancies because the risks of some adverse outcomes vary by parity [13, 14]. Observations with missing data on gestational age at birth among live born babies were excluded (*n* = 3). Ethical permission for this study was granted by the Regional Ethics Review Board in Stockholm (decision no. 2014/230–31, amendment no. 2020–00437).

### Maternal and infant outcomes

Using ICD-coded information from the MBR, the NPR and dispensation data from the Prescribed Drug Register, we investigated the following maternal outcomes: gestational diabetes, gestational hypertension, preeclampsia and eclampsia (diagnosed during pregnancy and up until discharge after delivery), placental abruption, postpartum hemorrhage, cesarean delivery, mode of vaginal delivery (operative vs. non-operative) and stillbirth. We also investigated the occurrence of the following outcomes during and within 3 months postpartum: infection, deep venous thrombosis and pulmonary embolism (VTE), cardiac arrest and maternal death (from the Cause of Death Register). Infant outcomes comprised preterm birth (< 37 weeks’ gestation), very preterm birth (< 32 weeks’ gestation), small or large for gestational age defined as ≥2 standard deviations (SD) below or above sex-specific mean weight per gestational age, respectively, Apgar score at 5 min less than 7, neonatal death (death < 27 days after birth) and infection within 3 months after birth. Major birth defects (non-chromosomal, based on EUROCAT coding [18] with modifications for Swedish coding [19]) were identified in the MBR and the NPR if an ICD code was received within 1 year of birth. ICD codes, ATC codes and data sources for each outcome are detailed in Additional file 1: Table S1.

### Other variables

Maternal weight and height (used to calculate body mass index (BMI)) and self-reported smoking status at first perinatal visit were obtained from the MBR. Pregestational diabetes and pregestational hypertension diagnosed before the last menstrual period were identified in the MBR, NPR and Prescribed Drug Register. Highest attained education at delivery was retrieved from the Education Register and used as a proxy for socioeconomic position during pregnancy. Maternal country of birth was retrieved from the Total Population Register. Data on pharmacologic treatments for sarcoidosis (prednisone, prednisolone, methotrexate, azathioprine and leflunomide) that were dispensed before, during or after pregnancy were identified from the Prescribed Drug Register for pregnancies with available prescription data (last menstrual period ≥ Oct 3, 2005 to allow for 3 months of dispensing data before conception).

### Statistical analysis

Baseline pregnancy characteristics were reported as either means with standard deviations or as proportions. Modified Poisson regression models were used to estimate relative risks (RR) and corresponding 95% confidence intervals (CI) of maternal and infant outcomes associated with sarcoidosis. In analyses of newborn outcomes, stillbirths were excluded. All models were adjusted for maternal age at delivery (continuous), calendar year of delivery (continuous) and educational level (< 9, 10–12, > 12, missing). If there were ≤ 10 events in the exposed or unexposed groups, models were only adjusted for age and year. In models for preeclampsia/eclampsia, preterm birth and major birth defects, we additionally adjusted for BMI (< 25, 25- < 30, ≥30 kg/m^2^, missing) and first trimester smoking status (yes, no, missing).

In a sensitivity analysis, we required only one visit listing sarcoidosis before pregnancy which increases power but may increase misclassification of sarcoidosis. To investigate the effect of missing data, missing values for BMI and smoking status were imputed with extreme values to assess their impact on the results. For example, in the case of smoking status, all patients with missing smoking information were first imputed to be smokers and in the following analysis assigned to be non-smokers.

For outcomes with fewer than five events, numbers were not reported and RRs were not estimated. Data was managed and analyzed using SAS software version 9.4 (SAS Institute Inc., Cary, NC, USA).

## Results

We identified 182 singleton first pregnancies in mothers with a history of a sarcoidosis diagnosis at conception and 6630 pregnancies in general population comparators without sarcoidosis between 2002 and 2013. Women with sarcoidosis were slightly older at delivery compared to women without sarcoidosis (32 vs. 31 years old) and were more likely to have a history of diabetes (2.2% vs. 1.2%) and hypertension (5.5% vs. 1.5%; Table 1). The median number of years since first diagnosis of sarcoidosis was 3.0 (interquartile range 1.4, 5.4).

**Table 1:** Characteristics of women with at least two sarcoidosis-coded visits before first pregnancy (*n* = 182) and general population comparators without sarcoidosis (*n* = 6630) in Sweden, 2002–2013

	Women with sarcoidosis *n* = 182	Women from the general population *n* = 6630
**Age at delivery in years, mean ± SD**	32.0 ± 4.5	30.9 ± 4.8
**Country of birth, n (%)**		
Nordic	169 (92.9)	5773 (87.1)
Non-Nordic	13 (7.1)	836 (12.6)
Missing	0 (0)	21 (0.3)
**Education, n (%)**		
≤ 9 years	8 (4.4)	399 (6.0)
10–12 years	61 (33.5)	2357 (35.6)
≥ 13 years	112 (61.5)	3734 (56.3)
Missing	1 (0.6)	140 (2.1)
**Body mass index at first prenatal visit, n (%)**		
Underweight (< 18.5 kg/m^2^)	3 (1.7)	128 (1.9)
Normal weight (18.5–24.9 kg/m^2^)	91 (50.0)	3851 (58.1)
Overweight (25.0–29.9 kg/m^2^)	50 (27.5)	1407 (21.2)
Obese (≥30 kg/m^2^)	16 (8.8)	576 (8.7)
Missing	22 (12.1)	668 (10.1)
**Smoking during first trimester, n (%)**		
Smoker	6 (3.3)	362 (5.5)
Non-Smoker	161 (88.5)	5917 (89.2)
Missing	15 (8.2)	351 (5.3)
**Pregestational diabetes**, n (%)	4 (2.2)	78 (1.2)
**Pregestational hypertension**, n (%)	10 (5.5)	96 (1.5)

There was no increased risk of gestational hypertension associated with sarcoidosis (RR 0.9; 95%CI 0.4, 2.1; Table 2). Nine percent of women with sarcoidosis had preeclampsia/eclampsia compared to 5% in the general population and the adjusted relative risk associated with sarcoidosis was 1.6 (95%CI 1.0, 2.6). The risk of cesarean delivery was increased by 30% in women with sarcoidosis (RR 1.3 95%CI 1.0, 1.6) and the increased risk was similar for elective and emergency cesarean deliveries. There was no increased risk of operative vaginal delivery nor postpartum hemorrhage. There were fewer than 5 cases of stillbirth, gestational diabetes and infection among women with sarcoidosis. There were no cases of maternal death, cardiac arrest, VTE and placental abruption.

**Table 2:** Maternal outcomes in first-time pregnancies with a history of at least two sarcoidosis-coded health care visits and general population comparator pregnancies in Sweden, crude and adjusted risk ratios with 95% confidence intervals, 2002–2013

	Sarcoidosis pregnancies (*n* = 182) n (%)	General population pregnancies (*n* = 6630) n (%)	Crude Risk Ratio (95% CI)	Adjusted Risk Ratio^a^ (95% CI)
**Antepartum**				
Gestational diabetes	< 5	71 (1.1)	NA	NA
Gestational hypertension	6 (3.3)	207 (3.1)	1.1 (0.5, 2.3)	0.9 (0.4, 2.1)
Preeclampsia/eclampsia	16 (8.8)	354 (5.3)	1.6 (1.0, 2.7)	1.6 (1.1, 2.6)
Stillbirth	< 5	26 (0.4)	NA	NA
**Delivery**				
Cesarean delivery	54 (29.7)	1414 (21.3)	1.4 (1.1, 1.7)	1.3 (1.0, 1.6)
Emergency	36 (19.8)	912 (13.8)	1.5 (1.1, 2.0)	1.4 (1.0, 1.9)
Elective	18 (9.9)	502 (7.6)	1.4 (0.9, 2.2)	1.3 (0.8, 2.0)
Operative-vaginal	25 (13.7)	923 (13.9)	1.1 (0.8, 1.6)	1.1 (0.7, 1.5)
Postpartum hemorrhage	13 (7.1)	404 (6.1)	1.2 (0.7, 2.0)	1.1 (0.6, 1.9)
Placental abruption	0 (0)	21 (0.3)	NA	NA
**Antepartum and postpartum (within 3 months)**				
Infection	< 5	207 (3.1)	NA	NA
Venous thromboembolism	0 (0)	17 (0.2)	NA	NA
Cardiac arrest	0 (0)	0 (0)	NA	NA
Maternal death	0 (0)	0 (0)	NA	NA

*NA* Not assessed if less than 5 cases to minimize identifiability of individuals

^a^Adjusted for maternal age, calendar year, and educational level. If there were ≤ 10 events in the exposed or unexposed groups, models were only adjusted for age and year. Preeclampsia/eclampsia was further adjusted for body mass index and smoking status

The risk of preterm delivery for sarcoidosis pregnancies was 70% higher than the risk in the general population (Table 3; RR 1.7; 95%CI 1.1, 2.5). There were fewer than five newborns who had very preterm birth, a 5-min Apgar score < 7 or were small or large for gestational age. There was no increased risk of infection within three months after birth (RR 0.9; 95%CI 0.5, 1.7) associated with maternal sarcoidosis. There were no neonatal deaths in the sarcoidosis group. 6.1% of sarcoidosis pregnancies had a major non-chromosomal birth defect compared to 3.7% in the general population (RR 1.6; 95%CI 0.9, 2.8). There was a similar percentage of cardiac birth defects in newborns of mothers with sarcoidosis and the general population (1.7% vs. 1.8%, respectively) but a higher percentage of non-cardiac birth defects (5.0% vs. 2.1%, respectively) which were mainly defects of the eye, ear, face, neck or digestive system. In a post-hoc analysis excluding women with pregestational and gestational diabetes, the results did not change.

**Table 3:** Infant outcomes in first-time pregnancies (excluding stillbirths) with a history of at least two sarcoidosis-coded health care visits (*n* = 181) and general population comparator pregnancies (*n* = 6604) in Sweden, crude and adjusted risk ratios, 2002–2013

	Sarcoidosis pregnancies n (%)	General population pregnancies n (%)	Crude Risk Ratio (95% CI)	Adjusted Risk Ratio^a^ (95% CI)
Preterm (< 37 wks gestation)	20 (11.1)	416 (6.3)	1.8 (1.1, 2.7)	1.7 (1.1, 2.5)
Very preterm (< 32 wks gestation)	< 5	52 (0.8)	NA	NA
Small Size for gestational age	< 5	188 (3.0)	NA	NA
Large Size for gestational age	< 5	102 (1.6)	NA	NA
Apgar at 5 min < 7	< 5	88 (1.3)	NA	NA
Major birth defect^b^	11 (6.1)	245 (3.7)	1.6 (0.9, 2.9)	1.6 (0.9, 2.8)
Infection (within 3 mo)^b^	10 (5.5)	369 (5.6)	1.0 (0.5, 1.8)	0.9 (0.5, 1.7)
Neonatal death	0 (0)	8 (0.1)	NA	NA

*NA* Not assessed if less than 5 cases to minimize identifiability of individuals

^a^Adjusted for maternal age, calendar year and educational level. The models for preterm birth and major birth defects were additionally adjusted for body mass index and smoking status

^b^Infants with a missing ID number were excluded due to inability to link to the patient register (*n* = 4, all excluded were general population comparator pregnancies)

**Fig. 1:**
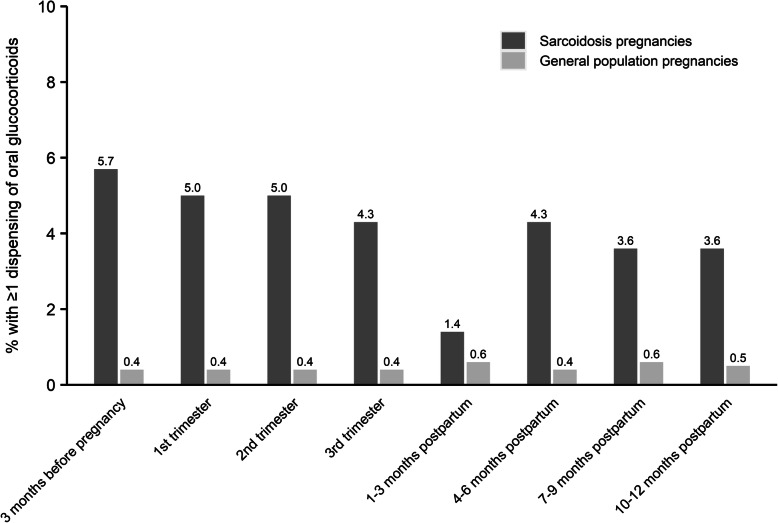
Proportion dispensed oral glucocorticoids in sarcoidosis (*n* = 140) and general population (*n* = 3441) pregnancies by trimester, 2006–2013

There were 140 sarcoidosis pregnancies and 3441 general population pregnancies with data on dispensed prescriptions between 3 months before and up to 1 year after pregnancy. There were no dispensations of azathioprine, methotrexate or leflunomide recorded < 3 months before or during any pregnancy with sarcoidosis. 7.9% of sarcoidosis pregnancies had at least one dispensation of oral glucocorticoids during pregnancy. In the three months before pregnancy, 5.7% of the sarcoidosis pregnancies had at least 1 dispensation of oral glucocorticoids. During the course of pregnancy, the proportion decreased to 4.3% in the 3rd trimester and was lowest in the 3 months after delivery (1.4%; Fig. 1).

When a less strict sarcoidosis definition was implemented, relative risks were similar to the main analysis (Additional file 2: Table S2). When missing values for the confounders BMI and smoking status were imputed with extreme values, results remained similar (Additional file 3**:** Table S3). In a post-hoc analysis, women were excluded who had a glucocorticoid dispensation < 9 months before LMP, a history of pregestational hypertension or diabetes (*n* = 29 women with sarcoidosis, *n* = 195 women without sarcoidosis) to determine whether these factors could explain the increased risks. The results were similar to the main results (Additional file 4; Table S4).

## Discussion

In this large population-based cohort study, sarcoidosis was associated with a 60% increased risk of preeclampsia/eclampsia and a 70% increased risk of preterm birth. Risk for cesarean delivery was 30% higher in pregnancies with sarcoidosis compared to the general population. We did not observe many severe complications during pregnancy or delivery for the mother. Newborns of mothers with sarcoidosis had an increased risk of birth defects, the majority of which were non-cardiac. In absolute numbers, most adverse outcomes in sarcoidosis pregnancies were uncommon, and the majority had uncomplicated pregnancies.

There were no cases of death, cardiac arrest, VTE or placental abruption linked to sarcoidosis. This is in contrast to a previous U.S. study which found a 6-fold increased risk of VTE [12]. The study from the U.S. also found a 70% increased risk of postpartum hemorrhage which we did not observe. Discrepancies in the relative risks between the two studies may be due to several differences. The study from the U.S. used data from the Healthcare Cost and Utilization Project Nationwide Inpatient Sample and identified sarcoidosis from delivery-related discharge diagnoses, potentially including women with a more active/severe disease at the time of delivery. In contrast, we identified sarcoidosis pregnancies by requiring at least two sarcoidosis health care visit *prior to* the pregnancy, which likely includes women with both active and resolved disease states. Definitions of pregnancy outcomes also vary between the two study populations. In Sweden, postpartum hemorrhage is defined as > 1000 ml blood loss (regardless of mode of delivery) compared to the WHO cut-off of > 500 ml [20]. There are also differences between the two countries in race, socioeconomic position and/or access to health care, which are related to disease severity and probably also adverse pregnancy outcomes [21]. The study from the U.S. was quite diverse, with Black Americans making up more than one third of the sarcoidosis cases. Furthermore, our study restricted to first births to account for differences in risk by parity because a selectively healthier group of women chose to have a subsequent pregnancy [13]. Despite these differences, we observed similarly increased risks of preeclampsia/eclampsia, cesarean delivery and preterm birth compared to the study from the U.S. Guidelines for care of mothers with sarcoidosis should address these risks, although they are not large; 9 out of 100 mothers with sarcoidosis had preeclampsia/eclampsia compared with 5 out of 100 mothers without sarcoidosis.

Newborns of women with sarcoidosis did not have more adverse outcomes compared to the general population in terms of size for gestational age, infection or neonatal death. However, we observed a higher risk of major birth defects associated with maternal sarcoidosis; 6.1% compared to 3.7% in the general population. There was no increased risk of cardiac birth defects, but an increased risk of non-cardiac birth defects. These congenital malformations might be more likely to be detected in infants born to women under surveillance for other comorbidities, thus causing a detection bias to inflate the risk in the sarcoidosis group. Another explanation for the higher risk of birth defects could be due to maternal characteristics such as diabetes or high BMI [19, 22], although we adjusted for BMI and excluded diabetes in a post-hoc analysis and results were similar. These results should be interpreted with caution due to the small number of events in the sarcoidosis group (*n* = 11).

Women with a history of sarcoidosis during pregnancy did not take many sarcoidosis-related medications; 7.9% were dispensed oral glucocorticoids at any time during pregnancy. Only 5.7% were dispensed oral glucocorticoids in the three months prior to pregnancy, indicating that few women had sarcoidosis in need of treatment. Interestingly, the lowest percentage of oral glucocorticoid use was in the first 3 months after delivery. This is consistent with some observations that sarcoidosis and other inflammatory diseases improve during pregnancy [6, 23, 24]. However, during the 4 to 6 months after delivery, the percentage increased again, potentially indicating a relapse. This should be investigated further in a larger study with information on disease activity.

Our findings are in line with studies in other inflammatory diseases which have shown an increased risk of preeclampsia, preterm birth and cesarean delivery compared to the general population [2–4]. The percent of first pregnancies affected by these outcomes in sarcoidosis are similar to those reported in inflammatory arthritis and inflammatory bowel disease in Sweden (5–6% preeclampsia, 8–11% preterm birth, 26–29% cesarean delivery) [3, 25, 26]. These highly intertwined outcomes have multiple causes which are difficult to disentangle in small samples [27]. Pregnancy involves a complex process of dynamic immune regulation, with an anti-inflammatory stage in the 2nd trimester and later a switch to a pro-inflammatory environment needed to initiate labor [28]. In sarcoidosis, the immune system’s ability to regulate and adapt to the immune environment during pregnancy may be impaired, leading to pregnancy complications. Comorbidities associated with sarcoidosis and its treatment may play a role in the pregnancy complications we observed in this study. However, when excluding women with glucocorticoid dispensations, hypertension or diabetes before pregnancy, the relative risks did not markedly change.

Sarcoidosis diagnosis and maternal and infant outcomes in this study were based on ICD codes, which may be misclassified. By restricting to at least two visits listing sarcoidosis, we aimed to minimize this bias. The use of nationwide registers allowed for increased power to study rare outcomes in this patient population, however we still had limited power for some outcomes and could not study mediating factors such as medication. An important limitation was the lack of information on disease activity or phenotype, which is likely related to pregnancy outcome. This should be addressed in future studies and used to identify women at the highest risk of adverse outcomes. Our results may not be generalizable to other populations with a different quality and accessibility of maternity care and/or different rates of cesarean delivery. A key strength of this study is the use of population-based Swedish national registers in a setting with universal access to healthcare. The use of Sweden’s Birth Register which captures 99% of all deliveries since 1973, made it possible to assess the parity-specific risk for pregnancy outcomes and to adjust for confounding variables, such as BMI at first prenatal visit and self-reported smoking status [15].

Mothers with sarcoidosis and their treating physicians should be aware of the increased risks of adverse pregnancy outcomes associated with sarcoidosis, although it should be kept in mind that these outcomes are uncommon. Early detection of these conditions may prevent potential adverse effects on mothers and newborns. Besides its immediate consequences, preeclampsia is a predictor for later development of cardiovascular disease [29]. This highlights the need for adequate follow-up care of women with sarcoidosis who have experienced pre-eclampsia.

To conclude, our findings demonstrate that sarcoidosis is associated with increased risks of preeclampsia/eclampsia, cesarean delivery, preterm delivery and non-cardiac birth defects. Awareness of these increased risks can be used for pregnancy planning and counselling for women with sarcoidosis.

## Supplementary information

**Additional file 1: Table S1.** Data sources, International classification of disease (ICD)-10 codes and anatomical therapeutic chemical classification (ATC) codes used to define maternal characteristics, and maternal and fetal pregnancy outcomes.

**Additional file 2: Table S2.** Maternal and infant outcomes in first-time pregnancies with at least one sarcoidosis-coded health care visit and general population comparator pregnancies in Sweden, crude and adjusted risk ratios with 95% confidence intervals, 2002–2013.

**Additional file 3: Table S3.** Sensitivity analysis with imputed extreme values of smoking and body mass index (BMI).

**Additional file 4: Table S4.** Maternal and infant outcomes in first-time pregnancies with at least two sarcoidosis-coded health care visit and general population comparator pregnancies in Sweden. Women were excluded who had a dispensation of oral glucocorticoids nine months before pregnancy, a history of diabetes or pregestational hypertension before pregnancy. Crude and adjusted risk ratios are presented with 95% confidence intervals, 2002–2013.

## Data Availability

The datasets used for the conduct of this study are covered by ethics and secrecy agreements and are not publicly available.

## Abbreviations

BMI: Body mass index
CI: Confidence interval
ICD: International Classification of Diseases
MBR: Medical Birth Register
NPR: National Patient Register
RR: Relative risk
SD: Standard deviation
VTE: Venous thromboembolism

## References

[1] Arkema EV, Grunewald J, Kullberg S, Eklund A, Askling J (2016). Sarcoidosis incidence and prevalence: a nationwide register-based assessment in Sweden. Eur Respir J.

[2] Wallenius M, Skomsvoll JF, Irgens LM, Salvesen KA, Nordvag BY, Koldingsnes W, Mikkelsen K, Kaufmann C, Kvien TK (2011). Pregnancy and delivery in women with chronic inflammatory arthritides with a specific focus on first birth. Arthritis Rheum.

[3] Remaeus K, Stephansson O, Johansson K, Granath F, Hellgren K (2019). Maternal and infant pregnancy outcomes in women with psoriatic arthritis: a Swedish nationwide cohort study. BJOG.

[4] Williams A, Grantz K, Seeni I, Robledo C, Li S, Ouidir M, Nobles C, Mendola P (2019). Obstetric and neonatal complications among women with autoimmune disease. J Autoimmun.

[5] Broms G, Granath F, Linder M, Stephansson O, Elmberg M, Kieler H (2014). Birth outcomes in women with inflammatory bowel disease: effects of disease activity and drug exposure. Inflamm Bowel Dis.

[6] Selroos O (1990). Sarcoidosis and pregnancy: a review with results of a retrospective survey. J Intern Med.

[7] Chapelon Abric C, Ginsburg C, Biousse V, Wechsler B, de Gennes C, Darbois Y, Janse Marec J, Godeau P, Piette JC (1998). Sarcoïdose et grossesse. Étude retrospective de 11 cas. La Revue de Médecine Interne.

[8] Maisel JA, Lynam T (1996). Unexpected sudden death in a young pregnant woman: unusual presentation of neurosarcoidosis. Ann Emerg Med.

[9] Reuhl J, Schneider M, Sievert H, Lutz FU, Zieger G (1997). Myocardial sarcoidosis as a rare cause of sudden cardiac death. Forensic Sci Int.

[10] Wallmuller C, Domanovits H, Mayr FB, Laggner AN (2012). Cardiac arrest in a 35-year-old pregnant woman with sarcoidosis. Resuscitation.

[11] Bobbak V, Mushlin N, Weibel S (2007). Sarcoidosis in pregnancy and postpartum period. Curr Resp Med Rev.

[12] Hadid V, Patenaude V, Oddy L, Abenhaim HA (2015). Sarcoidosis and pregnancy: obstetrical and neonatal outcomes in a population-based cohort of 7 million births. J Perinat Med.

[13] Hernandez-Diaz S, Toh S, Cnattingius S (2009). Risk of pre-eclampsia in first and subsequent pregnancies: prospective cohort study. BMJ.

[14] Shah PS (2010). Knowledge synthesis group on determinants of LBWPTb. Parity and low birth weight and preterm birth: a systematic review and meta-analyses. Acta Obstet Gynecol Scand.

[15] Cnattingius S, Ericson A, Gunnarskog J, Kallen B (1990). A quality study of a medical birth registry. Scand J Soc Med.

[16] Rossides M, Kullberg S, Askling J, et al. Sarcoidosis mortality in Sweden: a populationbased cohort study. Eur Respir J. 2018;51(2):1701815. 10.1183/13993003.01815-2017 PMC5886843 29467203

[17] Doyle P (1996). The outcome of multiple pregnancy. Hum Reprod.

[18] EUROCAT. EUROCAT guide 1.4. 2016 [cited 2019 September 4]; Available from: www.eurocat-network.eu/aboutus/datacollection/guidelinesforregistration/guide1_4.

[19] Persson M, Cnattingius S, Villamor E, Soderling J, Pasternak B, Stephansson O, Neovius M (2017). Risk of major congenital malformations in relation to maternal overweight and obesity severity: cohort study of 1.2 million singletons. BMJ.

[20] Dahlke JD, Mendez-Figueroa H, Maggio L, Hauspurg AK, Sperling JD, Chauhan SP, Rouse DJ (2015). Prevention and management of postpartum hemorrhage: a comparison of 4 national guidelines. Am J Obstet Gynecol.

[21] Rabin DL, Richardson MS, Stein SR, Yeager H (2001). Sarcoidosis severity and socioeconomic status. Eur Respir J.

[22] Bell R, Glinianaia SV, Tennant PW, et al. Peri-conception hyperglycaemia and nephropathy are associated with risk of congenital anomaly in women with pre-existing diabetes: a population-based cohort study. Diabetologia. 2012;55:936–47. 10.1007/s00125-012-2455-y 22314812

[23] de Man YA, Dolhain RJ, van de Geijn FE, Willemsen SP, Hazes JM (2008). Disease activity of rheumatoid arthritis during pregnancy: results from a nationwide prospective study. Arthritis Rheum.

[24] Gotestam Skorpen C, Lydersen S, Gilboe IM, Skomsvoll JF, Salvesen KA, Palm O, Koksvik HSS, Jakobsen B, Wallenius M (2017). Disease activity during pregnancy and the first year postpartum in women with systemic lupus Erythematosus. Arthritis Care Res (Hoboken).

[25] Norgaard M, Larsson H, Pedersen L, Granath F, Askling J, Kieler H, Ekbom A, Sorensen HT, Stephansson O (2010). Rheumatoid arthritis and birth outcomes: a Danish and Swedish nationwide prevalence study. J Intern Med.

[26] Broms G, Granath F, Linder M, Stephansson O, Elmberg M, Kieler H (2012). Complications from inflammatory bowel disease during pregnancy and delivery. Clin Gastroenterol Hepatol.

[27] Bandoli G, Singh N, Strouse J, Baer RJ, Donovan BM, Feuer SK, Nidey N, Ryckman KK, Jelliffe-Pawlowski LL, Chambers CD (2020). Mediation of adverse pregnancy outcomes in autoimmune conditions by pregnancy complications: a mediation analysis of autoimmune conditions and adverse pregnancy outcomes. Arthritis Care Res (Hoboken).

[28] Mor G, Aldo P, Alvero AB (2017). The unique immunological and microbial aspects of pregnancy. Nat Rev Immunol.

[29] Veerbeek JH, Hermes W, Breimer AY, van Rijn BB, Koenen SV, Mol BW, Franx A, de Groot CJ, Koster MP (2015). Cardiovascular disease risk factors after early-onset preeclampsia, late-onset preeclampsia, and pregnancy-induced hypertension. Hypertension.

