# Supplementary material for: Maternal and infant outcomes in sarcoidosis pregnancy: a Swedish population-based cohort study of first births
Source: Respir Res. 2020 Aug 27;21:225. doi: 10.1186/s12931-020-01493-y (PMC7457286; doi:10.1186/s12931-020-01493-y)
Supplement: Supplementary file 1 — Additional file 1: Table S1. Data sources, International classification of disease (ICD)-10 codes and anatomical therapeutic chemical classification (ATC) codes used to define maternal characteristics, and maternal and fetal pregnancy outcomes. [file 12931_2020_1493_MOESM1_ESM.docx]

**SUPPLEMENTARY INFORMATION**

**Table S1.** Data sources, International classification of disease (ICD)-10 codes and anatomical therapeutic chemical classification (ATC) codes used to define maternal characteristics, and maternal and fetal pregnancy outcomes.

|  | **Register source** | **ICD-10 codes and ATC codes, if applicable** | |
| --- | --- | --- | --- |
| Maternal variables |  |  |  |
| **Maternal characteristics** |  |  |  |
| Age at delivery | MBR |  |  |
| BMI at first prenatal visit | MBR |  |  |
| Smoking during early pregnancy | MBR |  |  |
| Country of birth of mother | Total Population Register |  |  |
| Parity | MBR |  |  |
| Living with partner | MBR |  |  |
| Years of education of mother | Education Register |  |  |
|  |  |  |  |
| **Pre-pregnancy comorbidities** |  |  |  |
| Pregestational diabetes | NPR and MBR and PDR |  | O24.0-O24.3, O24.8, E10-14  ATC: A10 |
| Pregestational hypertension | NPR, MBR and PDR |  | I10-15  ATC: C02CA, C07, C08, C09 |
| **Antepartum** |  |  |  |
| Gestational diabetes | NPR and MBR |  | O24.4 |
| Preeclampsia/eclampsia | NPR and MBR |  | O14-15 |
| Gestational hypertension | NPR and MBR |  | O13 |
| Placental abruption | NPR and MBR |  | O45 |
| Postpartum hemorrhage | NPR and MBR |  | O72.0-O72.3 |
|  |  |  |  |
| **Delivery** |  |  |  |
| Cesarean delivery (emergency, elective) | MBR |  |  |
| Vaginal delivery (non-operative, operative) | MBR |  |  |
| Onset of labor (induced, spontaneous) | MBR |  |  |
|  |  |  |  |
| **Antepartum and postpartum** |  |  |  |
| Cardiac arrest | NPR |  | I46 |
| Infection | NPR |  | A00-B99, G00-02, G042, G05-07,  H66-67, H70, J00-22, J32, J34.0, J36, J38.3, J39.0-39.1, K10.2, M00, M01, M46.2, M46.5, M86, N10, N30.0, L00-08 |
| Deep vein thrombosis | NPR and MBR |  | I81, I80.1-80.2, I82.2-82.9, O22.3, O22.8, O87.1, O87.9 |
| Pulmonary embolism | NPR and MBR |  | I26, O88.2 |
|  |  |  |  |
| **Prescribed medications for sarcoidosis treatment** | PDR |  | ATC: L01BA01, L04AX03, L04AA13, L04AX01, H02AB06, H02AB07 |

| Infant variables |  |  |  |
| --- | --- | --- | --- |
| Gestational age at birth | MBR |  |  |
| Apgar score at 5 minutes | MBR |  |  |
| Size for gestational age (small or large) | MBR |  |  |
| Neonatal death (0-27 days after birth) | MBR |  |  |
| Any major congenital anomaly, non-chromosomal | NPR and MBR |  | Q00-Q07, Q10.0, Q10.4, Q10.6-Q10.9, Q11-Q16 (excl Q13.5), Q17.6-Q17.8, Q18.3, Q18.8, Q20-Q25, (if preterm and received codes Q25.0 or Q25.6, it was not considered a malformation) Q26.0, Q26.2-Q26.9,Q30.0, Q32.1-Q32.9, Q33.0, Q33.2-Q33.5, Q33.7-Q33.9, Q34-Q37, Q38.0, Q38.3-Q38.9, Q39, Q40.2-Q40.9, Q41-Q42, Q43.1-Q43.9, Q44-Q45, Q50-Q51, Q52.0-Q52.2, Q52.4, Q52.6, Q52.8, Q52.9, Q54-Q56, Q60, Q61.1-Q61.9, Q62.0-Q62.6, Q62.8, Q62.9, Q63.0-Q63.2, Q63.4-Q63.9, Q64, Q66.0, Q66.1, Q67.9, Q68.1, Q68.2, Q68.6-Q68.9, Q70-Q74, Q75.0, Q77, Q78.2-Q78.8, Q79.0, Q79.2-Q79.5, Q80, Q81, Q82.0-Q82.4, Q82.6-Q82.9, Q86.0, Q89.0, Q89.3, Q89.4 |
|  |  |  |  |
| Infection | NPR |  | A00-B99, G00-02, G042, G05-07,  H66-67, H70, J00-22, J32, J34.0, J36, J38.3, J39.0-39.1, K10.2, M00, M01, M46.2, M46.5, M86, N10, N30.0, L00-08 |

NPR: National Patient Register – inpatient component 1987-2013, outpatient component 2001-2013; MBR: Medical Birth Register 1973-2013; PDR: Prescribed Drug Register 2005-2015; Education Register 1990-2013; Death Register 1961-2015; Total Population Register 1971-2014: ICD: International Classification of Diseases; ATC: Anatomical Therapeutic Chemical Classification
