# Supplementary material for: Maternal and infant outcomes in sarcoidosis pregnancy: a Swedish population-based cohort study of first births
Source: Respir Res. 2020 Aug 27;21:225. doi: 10.1186/s12931-020-01493-y (PMC7457286; doi:10.1186/s12931-020-01493-y)
Supplement: Supplementary file 2 — Additional file 2: Table S2. Maternal and infant outcomes in first-time pregnancies with at least one sarcoidosis-coded health care visit and general population comparator pregnancies in Sweden, crude and adjusted risk ratios with 95% confidence intervals, 2002–2013. [file 12931_2020_1493_MOESM2_ESM.docx]

**SUPPLEMENTARY INFORMATION**

**Table S2.** Maternal and infant outcomes in first-time pregnancies with at least one sarcoidosis-coded health care visit and general population comparator pregnancies in Sweden, crude and adjusted risk ratios with 95% confidence intervals, 2002-2013.

|  | **Sarcoidosis pregnancies**  (n= 259)  n (%) | **General population pregnancies**  (n= 6,630)  n (%) | **Crude  Risk Ratio** (95% CI) | **Adjusted  Risk Ratio*** (95% CI) |
| --- | --- | --- | --- | --- |
| **Maternal Outcomes** |  |  |  |  |
| **Antepartum** |  |  |  |  |
| Gestational diabetes | NA | 71 (1.1) | NA | NA |
| Gestational hypertension | 10 (3.9) | 207 (3.1) | 1.2 (0.7, 2.3) | 1.1 (0.6, 2.1) |
| Preeclampsia/eclampsia | 23 (8.9) | 354 (5.3) | 1.7 (1.1, 2.5) | 1.6 (1.1, 2.4) |
| Infection | NA | 166 (2.5) | NA | NA |
| Stillbirth | NA | 26 (0.4) | NA | NA |
| **Delivery** |  |  |  |  |
| Cesarean delivery | 73 (28.2) | 1,414 (21.3) | 1.3 (1.1, 1.6) | 1.2 (1.0, 1.5) |
| Emergency | 48 (18.5) | 912 (13.8) | 1.4 (1.1, 1.8) | 1.3 (1.0, 1.7) |
| Elective | 25 (9.7) | 502 (7.6) | 1.3 (0.9, 2.0) | 1.2 (0.8, 1.8) |
| Operative-vaginal | 36 (14.0) | 923 (13.9) | 1.1 (0.8, 1.5) | 1.1 (0.8, 1.4) |
| Postpartum hemorrhage | 16 (6.2) | 404 (6.1) | 1.0 (0.6, 1.7) | 1.0 (0.6, 1.6) |
| Placental abruption | 0 (0) | 21 (0.3) | NA | NA |
| **Antepartum and postpartum (within 3 months)** |  |  |  |  |
| Infection | NA | 41 (0.6) | NA | NA |
| Venous thromboembolism | NA | 17 (0.2) | NA | NA |
| Cardiac arrest | 0 (0) | 0 (0) | NA | NA |
| Maternal death | 0 (0) | 0 (0) | NA | NA |
| **Infant Outcomes** |  |  |  |  |
| Preterm (<37 wks gestation) | 31 (12.0) | 416 (6.3) | 1.9 (1.4, 2.7) | 1.8 (1.3, 2.5) |
| Very preterm (<32 wks gestation) | 5 (1.9) | 52 (0.8) | 2.5 (1.0, 6.1) | 2.4 (1.0, 6.0) |
| Small Size for gestational age | 6 (2.6) | 188 (3.0) | 0.9 (0.4, 1.9) | 0.9 (0.4, 1.9) |
| Large Size for gestational age | NA | 102 (1.6) | NA | NA |
| Apgar at 5 min <7 | NA | 88 (1.3) | NA | NA |
| Major birth defect** | 17 (6.6) | 245 (3.7) | 1.8 (1.1, 2.9) | 1.8 (1.1, 3.0) |
| Infection (within 3 mo)** | 14 (5.4) | 369 (5.6) | 1.0 (0.6, 1.7) | 0.9 (0.5, 1.6) |
| Neonatal death | 0 (0) | 8 (0.1) | NA | NA |

NA: Not assessed if less than 5 cases to minimize identifiability of individuals.

*Adjusted for maternal age, calendar year, and educational level. If there were ≤10 events in the exposed or unexposed groups, models were only adjusted for age and year. Models for preeclampsia/eclampsia, preterm birth and major birth defects were further adjusted for body mass index and smoking status.

**infants with a missing ID number were excluded due to inability to link to the patient register (n=4, all were general population comparator pregnancies)
