# Supplementary material for: Maternal and infant outcomes in sarcoidosis pregnancy: a Swedish population-based cohort study of first births
Source: Respir Res. 2020 Aug 27;21:225. doi: 10.1186/s12931-020-01493-y (PMC7457286; doi:10.1186/s12931-020-01493-y)
Supplement: Supplementary file 3 — Additional file 3: Table S3. Sensitivity analysis with imputed extreme values of smoking and body mass index (BMI). [file 12931_2020_1493_MOESM3_ESM.docx]

**SUPPLEMENTARY INFORMATION**

**Table S3.** Sensitivity analysis with imputed extreme values of smoking and body mass index (BMI).

| Outcomes | **Primary analysis RR (95% CI)**  (indicator for missing values) | **Adjusted RR** (95% CI) SINGLE IMPUTATION lowest value BMI | **Adjusted RR** (95% CI) SINGLE IMPUTATION highest value BMI | **Adjusted RR** (95% CI) SINGLE IMPUTATION lowest value smoking | **Adjusted RR** (95% CI) SINGLE IMPUTATION highest value smoking |
| --- | --- | --- | --- | --- | --- |
| Preeclampsia/eclampsia | 1.6 (1.1, 2.6) | 1.6 (1.0, 2.6) | 1.6 (1.0, 2.6) | 1.6 (1.0, 2.6) | 1.6 (1.0, 2.6) |
|  |  |  |  |  |  |
| Cesarean delivery | 1.3 (1.0, 1.6) | 1.3 (1.0, 1.6) | 1.3 (1.0, 1.6) | 1.3 (1.0, 1.6) | 1.3 (1.0, 1.6) |
|  |  |  |  |  |  |
| Preterm (< 37 weeks) | 1.7 (1.3, 2.5) | 1.7 (1.1, 2.5) | 1.7 (1.1, 2.5) | 1.7 (1.1, 2.6) | 1.7 (1.1, 2.5)) |
|  |  |  |  |  |  |
| Major birth defect | 1.6 (0.9, 2.8) | 1.6 (0.9, 2.8) | 1.6 (0.9, 2.8) | 1.6 (0.9, 2.8) | 1.6 (0.9, 2.8) |
