# Supplementary material for: Maternal and infant outcomes in sarcoidosis pregnancy: a Swedish population-based cohort study of first births
Source: Respir Res. 2020 Aug 27;21:225. doi: 10.1186/s12931-020-01493-y (PMC7457286; doi:10.1186/s12931-020-01493-y)
Supplement: Supplementary file 4 — Additional file 4: Table S4. Maternal and infant outcomes in first-time pregnancies with at least two sarcoidosis-coded health care visit and general population comparator pregnancies in Sweden. Women were excluded who had a dispensation of oral glucocorticoids nine months before pregnancy, a history of diabetes or pregestational hypertension before pregnancy. Crude and adjusted risk ratios are presented with 95% confidence intervals, 2002–2013. [file 12931_2020_1493_MOESM4_ESM.docx]

**SUPPLEMENTARY INFORMATION**

**Table S4.** Maternal and infant outcomes in first-time pregnancies with at least two sarcoidosis-coded health care visit and general population comparator pregnancies in Sweden. Women were excluded who had a dispensation of oral glucocorticoids nine months before pregnancy, a history of diabetes or pregestational hypertension before pregnancy. Crude and adjusted risk ratios are presented with 95% confidence intervals, 2002-2013.

|  | **Sarcoidosis pregnancies**  (n=153)  n (%) | **General population pregnancies**  (n= 6,433)  n (%) | **Crude  Risk Ratio** (95% CI) | **Adjusted  Risk Ratio*** (95% CI) |
| --- | --- | --- | --- | --- |
| **Maternal Outcomes** |  |  |  |  |
| **Antepartum** |  |  |  |  |
| Gestational diabetes | NA | 53 (0.8) | NA | NA |
| Gestational hypertension | 5 (3.3) | 192 (3.0) | 1.1 (0.5, 2.6) | 1.0 (0.4, 2.4) |
| Preeclampsia/eclampsia | 13 (8.5) | 324 (5.0) | 1.7 (1.0, 2.9) | 1.6 (1.0, 2.8) |
| Infection | NA | 160 (2.5) | NA | NA |
| Stillbirth | NA | 24 (0.4) | NA | NA |
| **Delivery** |  |  |  |  |
| Cesarean delivery | 40 (26.1) | 1,341 (20.9) | 1.3 (1.0, 1.6) | 1.2 (0.9, 1.6) |
| Emergency | 28 (18.3) | 871 (13.5) | 1.4 (1.0, 1.9) | 1.3 (0.9, 1.8) |
| Elective | 12 (7.8) | 470 (7.3) | 1.1 (0.7, 2.0) | 1.1 (0.6, 1.8) |
| Operative-vaginal | 20 (13.0) | 902 (14.0) | 1.0 (0.7, 1.5) | 1.0 (0.7, 1.5) |
| Postpartum hemorrhage | 10 (6.5) | 391 (6.1) | 1.1 (0.6, 2.0) | 1.0 (0.6, 1.9) |
| Placental abruption | 0 (0) | 20 (0.3) | NA | NA |
| **Antepartum and postpartum (within 3 months)** |  |  |  |  |
| Infection | 0 (0) | 38 (0.6) | NA | NA |
| Venous thromboembolism | NA | 17 (0.3) | NA | NA |
| Cardiac arrest | 0 (0) | 0 (0) | NA | NA |
| Maternal death | 0 (0) | 0 (0) | NA | NA |
| **Infant Outcomes** |  |  |  |  |
| Preterm (<37 wks gestation) | 13 (8.6) | 392 (6.1) | 1.4 (0.8, 2.4) | 1.3 (0.8, 2.3) |
| Very preterm (<32 wks gestation) | NA | 48 (0.8) | NA | NA |
| Small Size for gestational age | NA | 179 (2.9) | NA | NA |
| Large Size for gestational age | NA | 93 (1.5) | NA | NA |
| Apgar at 5 min <7 | NA | 87 (1.4) | NA | NA |
| Major birth defect** | 9 (5.9) | 234 (3.7) | 1.6 (0.8, 3.1) | 1.6 (0.8, 3.0) |
| Infection (within 3 mo)** | 7 (4.6) | 357 (5.6) | 0.8 (0.4, 1.7) | 0.8 (0.4, 1.7) |
| Neonatal death | 0 (0) | 8 (0.1) | NA | NA |

NA: Not assessed if less than 5 cases to minimize identifiability of individuals.

*Adjusted for maternal age, calendar year, and educational level. If there were ≤10 events in the exposed or unexposed groups, models were only adjusted for age and year. Models for preeclampsia/eclampsia, preterm birth and major birth defects were further adjusted for body mass index and smoking status.

**infants with a missing ID number were excluded due to inability to link to the patient register (n=4, all were general population comparator pregnancies)
